# Separate Coding of Different Gaze Directions in the Superior Temporal Sulcus and Inferior Parietal Lobule

**DOI:** 10.1016/j.cub.2006.10.052

**Published:** 2007-01-09

**Authors:** Andrew J. Calder, John D. Beaver, Joel S. Winston, Ray J. Dolan, Rob Jenkins, Evelyn Eger, Richard N.A. Henson

**Affiliations:** 1 Medical Research Council, Cognition and Brain Sciences Unit, 15 Chaucer Road, CB2 7EF Cambridge, United Kingdom; 2 Wellcome Department of Imaging Neuroscience, 12 Queen Square, London, WC1N 3BG, United Kingdom

**Keywords:** SYSNEURO

## Abstract

Electrophysiological recording in the anterior superior temporal sulcus (STS) of monkeys has demonstrated separate cell populations responsive to direct and averted gaze 1, 2. Human functional imaging has demonstrated posterior STS activation in gaze processing, particularly in coding the intentions conveyed by gaze 3, 4, 5, 6, but to date has provided no evidence of dissociable coding of different gaze directions. Because the spatial resolution typical of group-based fMRI studies (∼6–10 mm) exceeds the size of cellular patches sensitive to different facial characteristics (1–4 mm in monkeys), a more sensitive technique may be required. We therefore used fMRI adaptation, which is considered to offer superior resolution [7], to investigate whether the human anterior STS contains representations of different gaze directions, as suggested by non-human primate research. Subjects viewed probe faces gazing left, directly ahead, or right. Adapting to leftward gaze produced a reduction in BOLD response to left relative to right (and direct) gaze probes in the anterior STS and inferior parietal cortex; rightward gaze adaptation produced a corresponding reduction to right gaze probes. Consistent with these findings, averted gaze in the adapted direction was misidentified as direct. Our study provides the first human evidence of dissociable neural systems for left and right gaze.

## Results and Discussion

**Figure 1:**
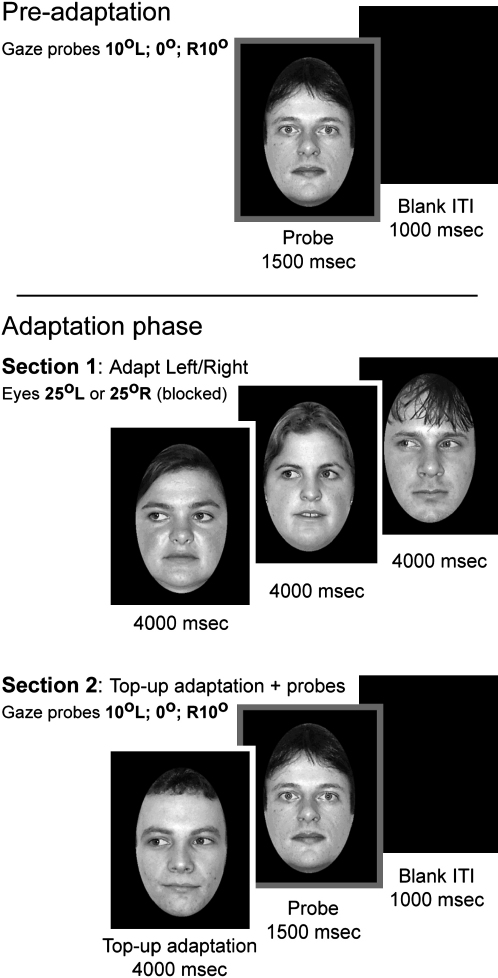
Experimental Design and Example Stimuli The adaptation experiment comprised five sections—an initial preadaptation phase to familiarize the subjects with probe stimuli and task and four adaptation phases comprising two sections each. The format of the trials in each section is illustrated in Figure 1. The preadaptation phase comprised four presentations of the 12 models (six male and six female) posing three gaze directions (10° left, 0° (direct), and 10° right; 144 stimuli in total). Trials consisted of a 1500 ms. presentation of a probe face and then a blank intertrial interval (ITI) of 1000 ms. Presentation order was randomized, and subjects pressed one of three keys with their right hand to indicate gaze direction. There were four adaptation phases, ordered either as LRRL or RLLR (where L = left adaptation and R = right adaptation) and counterbalanced across subjects. The second and third phases were separated by short breaks. Each phase had the same basic structure and comprised two sections. Section 1 contained two 4000 ms presentations of each of the 12 models gazing in one consistent direction—25° left or 25° right). Subjects were instructed to stare at the eye region of each face, and no response was required. Trials in section 2 consisted of a “top-up” adaptation face (4000 ms) gazing 25° in the adapted direction (i.e., same direction as in section 1) and, immediately after this, a probe face (1500 ms), and then a blank ITI (1000 ms). Probes were identical to those from the Pre-adaptation phase (12 models × three gaze directions [10° left, 0°, and 10° right] × two presentations; 72 stimuli in total), and subjects categorized their gaze as left, direct, or right. The top-up adaptation and probe faces were of similar size and were shown in the same central position, but they never had the same identity. In addition, vertical eye position and interocular distance were deliberately not standardized across identities to ensure that switching between the top-up and probe faces did not induce perception of apparent gaze motion [4]. Probe faces were always identified with a bold outline as illustrated. All images were 256 grayscale. The whole experiment lasted just over 1 hr.

Our fMRI-adaptation paradigm used a modified version of a recent behavioral experiment [8], which showed that adapting to a series of faces gazing left caused subjects to misidentify leftward gaze as direct; similarly, rightward gaze adaptation produced a corresponding pattern. The experiment comprised five distinct phases—an initial “pre-adaptation” gaze-detection phase and four adaptation phases that used the same “adapt and top-up” design (Figure 1). Subjects performed a gaze-discrimination task (indicating leftward, direct, or rightward gaze).

FMRI adaptation is believed to have an advantage over standard fMRI paradigms because it has the potential to demonstrate separate cell populations tuned to different stimulus categories (A and B), even if they are intermixed within the same imaging voxel [9]. Evidence for separable coding is found if repetitions of stimulus A (or B) produce a decreased BOLD response relative to intermixed presentations of different stimuli 10, 11. Using this procedure, research has shown that the posterior fusiform gyrus shows adaptation to repetitions of the same facial image across variations in retinal size 7, 12 and to the same facial identity across different expressions [13], supporting this region's posited role in facial-identity recognition [14].

Our hypothesis related to changes in activation to left and right gaze probes as a function of left and right adaptation. We predicted that if these different gaze directions are coded by separate STS cell populations, then adapting to one or the other gaze direction should produce reduced activation to probe faces gazing in the congruent direction (e.g., adapt left-probe left) relative to probes with incongruent gaze (e.g., adapt left-probe right). Moreover, if the neural representation of different gaze directions in humans is anatomically homologous to that in monkeys, then the anterior STS should show an effect of adaptation. Alternatively, if the posterior STS region identified by earlier fMRI studies of gaze processing in humans 3, 4 underlies the perceptual representation of gaze, as opposed to another aspect of gaze processing, then this area should be highlighted.

Parallel effects were predicted for behavioral and imaging data, so both were analyzed with the same statistical contrasts. These compared trials in which the gaze direction of adaptation and probe faces was incongruent (i.e., 25° left [or right] adaptation followed by 10° right [or left] probe face) relative to trials in which they were congruent (i.e., 25° left [or right] adaptation followed by 10° left [or right] probe face). Because the direction of the effect was predicted (i.e., reduced activation/sensitivity for the congruent relative to incongruent [or direct] condition), one-tailed tests were appropriate throughout. The same data sets were also analyzed by ANOVA. This confirmed the behavioral adaptation and identified brain regions identical to those found with the reported contrasts (see Supplemental Data available online).

### Behavior

**Figure 2:**
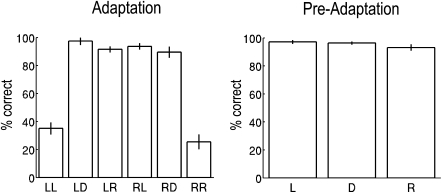
Behavioral Data Mean percentage of correct gaze responses to probe faces (10° left, direct, and 10° right) as a function of the direction of gaze adaptation (left and right). Performance for the same probe faces in the preadaptation phase is also shown for comparison. Error bars show standard errors. For the adaptation graph, LL = left adaptation-left gaze probe, LD = left adaptation-direct gaze probe, and so on. For the preadaptation data (right graph), L = left probe, D = direct probe, and R = right probe.

Figure 2 summarizes subjects' correct responses after leftward and rightward adaptation; performance for the preadaptation (no adaptation) phase is also shown for comparison. Prior to adaptation, subjects were accurate at discerning direct (96% correct) and 10° left/right averted gaze (95% correct). Adapting to 25° right or left gaze produced a striking reduction in correct detection of 10° gaze in the congruent direction relative to 10° gaze in the incongruent direction (t(13) = 12.14, p < 0.0001). Congruent and direct gaze probes also differed (t(13) = 9.14, p < 0.0001), whereas the two “nonadapted” conditions did not (direct versus incongruent, t < 1). Supplemental Data show that adaptation caused subjects to misidentify gaze in the adapted direction as “direct.”

### Superior Temporal Sulcus

**Figure 3:**
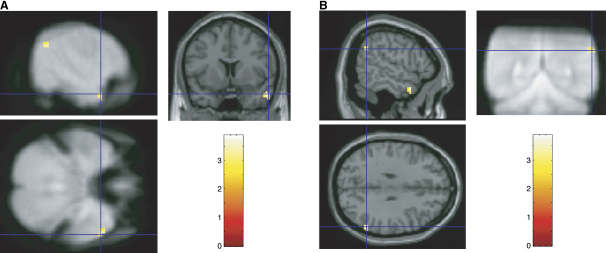
Sagittal, Coronal, and Transverse Slices through the Anterior STS and IPL (A) The right anterior superior temporal sulcus (57, 9, −27): Sagittal and transverse sections on the mean across subjects of their normalised mean EPI image, and a coronal section of a canonical T1-weighted image (both in MNI space). (B) The right inferior parietal lobule (60, −54, 30): Sagittal and transverse sections on a canonical T1-weighted image in MNI space and a coronal section of the mean, across subjects, of their normalized mean EPI image in MNI space. Both are thresholded at p < .005 (5 contiguous voxels) for purposes of illustration.

**Figure 4:**
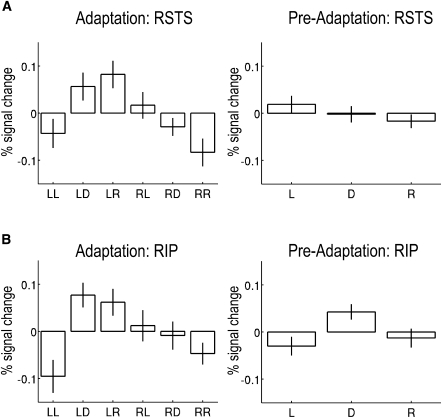
Neuroimaging Data Mean event-related response to each of the three types of probe faces (10° left, direct, and 10° right) as a function of the direction of gaze adaptation (left and right) for (A) the maximally activated voxel in the right anterior STS (RSTS; 57, 9, −27) and (B) right inferior parietal lobule (RIP; 60, −54, 30). Also shown for the same voxels is the mean event-related response to the same probe faces in the preadaptation phase. The *y* axis represents estimated peak percent signal change relative to the average over all voxels and scans; error bars show standard error of the mean, between-subject differences having been removed. For the adaptation data (left graphs), LL = left adaptation-left gaze probe; LD = left adaptation-direct gaze probe, and so on. For the preadaptation data (right graphs), L = left probe, D = direct probe, and R = right probe.

After preprocessing, we estimated the event-related response to each of the three types of probe faces (left, direct, and right gaze) as a function of the adapting gaze direction (left or right). A contrast, which recapitulated that used for the behavioral data, compared trials on which the direction of gaze of the adaptation and probe stimuli were congruent relative to trials on which they were incongruent. Only two brain regions, implicated in gaze perception and orientation of attention, showed a significant effect across the entire brain. One area was the anterior superior temporal sulcus (STS) (57, 9, −27, T = 3.87 (Z = 3.66), p < 0.001) (Figures 3A and 4A). Further analysis of the maximally activated voxel's data showed that congruent (adapted) and direct gaze probes differed (t(13) = −2.2, p = 0.023), whereas the two “nonadapted” conditions did not (direct versus incongruent, t(13) = −1.24, p = 0.24). Figure 4A shows that the mean event-related responses to left, direct, and right probes for this same voxel in the preadaptation phase were statistically equivalent (F < 1). Thus, the pattern we have observed after adaptation clearly reflects the influence of the adapting gaze stimulus.

The involvement of the anterior STS parallels research showing that this region codes different gaze directions in monkeys 1, 2, 15. However, whereas the only averted-gaze cells identified by nonhuman primate research produced a “symmetric” response to both left *and* right gaze, our current study provides the first evidence of selective coding of these gaze directions.

More recent research with macaques has shown that face cells in an STS region anatomically homologous to the one identified here (i.e., the rostral anterior STS) were more likely to be modulated by different gaze directions (82% of cells) than were cells in an adjacent posterior section (45%) [15]. Furthermore, the same region contained the largest proportion of “asymmetric” cells with different response patterns to rightward and leftward head and gaze combinations. Asymmetric coding of gaze alone as reported here (i.e., frontal face with left or right gaze) was not shown, although this was not the focus of the study; see also 1, 2 for symmetric and asymmetric coding of head direction.

### Inferior Parietal Lobule

The second brain region identified in the above contrast was the right inferior parietal lobule IPL (60, −54, 30, T = 3.52 (Z = 3.36), p < 0.001) (Figures 3B and 4B). The pattern of activation was similar to that of the STS, and once again, further analysis of the maximal voxel's data showed that congruent (adapted) and direct probes also differed (t(13) = -2.8, p = 0.007), whereas the incongruent and direct conditions did not (t < 1). An analysis of the mean event-related response to each probe face in the preadaptation phase for the same voxel (Figure 4B) showed a significant difference between probes for direct gaze and those for both left and right gaze (13) = 2.64, p = 0.02); however, left and right conditions were statistically equivalent (t < 1). Thus, again, the significant incongruent versus congruent contrast reflects the influence of the adapting stimulus.

Earlier functional imaging research has identified a role for the IPL in gaze processing 5, 6, 16, and it is of note that it is connected to different areas of the STS 17, 18. In addition, recent cell recording in macaques has identified inferior parietal cells (area LIP) responsive to gaze cues (Deaner, R.O., Klein, J.T., and Platt, M.L., Society for Neuroscience, 2005). These findings may reflect the more general role of parietal cortex in orienting attention 19, 20, 21, which is initiated by viewing averted gaze 22, 23, 24, and more specifically the role of the right inferior parietal cortex (as a component of the ventral frontoparietal attentional network) in reorienting attention toward behaviorally relevant events [25]. Hence, this region's involvement may reflect adaptation of attentional orienting mechanisms rather than perceptual representations of gaze. Because our acquisition sequence was optimized to identify activation in temporal cortex, it did not include the superior parietal regions. Hence, it is possible that adaptation of gaze perception may affect the dorsal intraparietal sulcus, which has also been activated in fMRI studies of gaze perception 3, 4.

### Right Lateralization of the Effects

Because suprathreshold voxels in the STS and IPL regions were restricted to the right hemisphere, we formally tested for laterality effects by examining responses of homologous regions in the left hemisphere (by inverting the sign of the maximum voxel's x coordinate). The left STS voxel showed a pattern that was similar, although statistically subthreshold, to the right; no discernible pattern was present for the left IPL voxel (see Supplemental Data). We entered the “congruency” effect (i.e., the contrast of conditions tested above) from all four voxels into a 2 × 2 ANOVA examining hemisphere (left, right) and region (STS, IPL). This showed a significant main effect of hemisphere (F(1,13) = 9.75, p = 0.008) but no effect of region nor interaction (Fs <1), confirming a right lateralization of the effects. Further inspection showed no left-hemisphere activation even at a reduced threshold (p < 0.05, uncorrected).

The right lateralization may simply reflect the predominant role of this hemisphere in face perception 14, 26. In addition, although bilateral STS activation was found in earlier studies of gaze perception 3, 4, more recent work has shown mainly right activation 5, 6, 27, extending into the IPL. It is also relevant that the ventral frontoparietal attentional network is predominantly lateralized to the right [25].

### STS Function in Gaze Perception

Previous functional-imaging studies using standard imaging paradigms in humans have emphasized the role of the *posterior* STS in different gaze-processing tasks 3, 4, 5, 27. This research has found no clear preference for a particular gaze direction, with different studies showing maximal posterior STS activation to averted gaze 3, 4, maximal activation to mutual gaze [6], equivalent activation to both [16], or no STS activation 28, 29. The contribution of this region to gaze processing is informed by recent research 6, 27 showing that it is particularly sensitive to the “intentionality” signaled bygaze (i.e., we look at objects toward which we have some intention—objects that we might approach or grasp) rather than the perceptual structure of gaze per se (see also [30]). Hence, the idea that intentionality can differ with experimental context may explain the variation in relative activation for different gaze directions in the posterior STS. For example, in one study subjects saw a virtual-reality human figure approach them and then gaze directly at them; repositioning the same figure caused it to look away from the subjects [6]. Despite the fact that both conditions contained the same physical stimulus, significantly increased posterior STS activation was observed for the mutual-gaze version. A second study demonstrated that the posterior STS is particularly sensitive to violation of “expected intentionality” signaled by *averted* gaze and that this effect is absent in autistic individuals 5, 27, who are known to show impaired interpretation of the goals and intentions of others but preserved perception of gaze direction 31, 32.

Together, these studies suggest that the posterior STS is sensitive to the intentionality of gaze and other biological signals [33] (see also [34] for evidence in the macaque), whereas our current results and cell recording in monkeys implicate the anterior STS in separable coding of different gaze directions.

Finally, given that different gaze angles were used as the adaptation (25° left/right) and probe stimuli (10° left/right), our effects are best interpreted as adaptation of leftward and rightward gaze as opposed to adaptation of a specific gaze angle or stimulus configuration (e.g., 10° versus 25° left). Similarly, for cells responsive to head direction (which has been studied more extensively than gaze), the preferred direction clusters around a limited number of prototypical views (i.e., left, right, upward, downward, etc.), as opposed to more finely tuned directions [35]. Two further points are of note. First, different people's faces were used in the “top-up” and probe face events of the adaptation trials (Figure 1), hence, the effects cannot reflect adaptation of facial identity, which previous research has attributed to the posterior fusiform gyrus 7, 12, 13, 36. Second, although the top-up and probe faces in this study were of similar size, our previous behavioral work shows that gaze adaptation persists across changes in retinal size [8].

In conclusion, our study provides the first human evidence that left and right gaze directions are coded by distinct neuronal populations in the right anterior STS. A similar dissociation was found in the right IPL, and we have attributed this dissociation to that region's role in attentional orienting. A parallel analysis of the behavioral data produced a complementary pattern, with decreased sensitivity to left and right gaze on congruent relative to incongruent trials. Together with other fMRI-adaptation research 7, 13, our study supports the componential nature of face perception with different functional and neural mechanisms underlying the representation of gaze (STS) and facial identity (posterior fusiform gyrus) [14]. Of more significance, our present results demonstrate that the functional basis of human face perception requires further fractionation, with dissociable neural mechanisms underlying the perception and attentional processing of different gaze directions.

## Experimental Procedures

### Subjects

Sixteen right-handed healthy volunteers with normal vision completed the study for payment. The study was approved by a local ethics committee, and subjects provided written informed consent. Two subjects' data were discarded because of equipment failure, leaving seven females and seven males, mean age = 22.7 years (SD = 3.3).

Materials and design are summarized in Figure 1.

### fMRI Acquisition and Analysis

See Supplemental Data for details of fMRI acquisition and data analysis. In brief, BOLD-weighted echoplanar (EPI) images of 32 near-transverse slices (3 × 3 × 3 mm^3^ voxels) were acquired with a repetition time (TR) of 2080 ms. After realignment, normalization, and spatial smoothing (8 mm), the images were analyzed with the general linear model with SPM2 (www.fil.ion.ucl.ac.uk/spm2.html). Correct and incorrect responses to all experimental trials were modeled by delta functions convolved with a canonical hemodynamic response function (HRF). Maximum likelihood estimates of the parameters of the resulting regressors were obtained with a temporal high-pass filter (cut-off 128 s) and an AR(1) model of temporal autocorrelation. Contrasts of these parameter estimates were entered into a second-level “group” analysis, in which subjects were the only random effect. For the adaptation phase, this analysis included the six conditions of interest (two adapted directions × three probe directions), within which the critical, directional interaction between adapting gaze direction and probe gaze direction was evaluated as a T contrast, creating a statistical parametric map (SPM) of the T statistic. Effects were predicted for regions implicated in gaze perception, specifically the STS and parietal cortex 1, 2, 3, 4, 5, 6, 15, 16, 37. Because no standard anatomical ROIs are available for both a priori regions, we thresholded the SPMs at an a priori threshold of p < 0.001, uncorrected for multiple comparisons.

## Supplemental Data

Document S1. Supplemental Results and One Table
