# Supplementary material for: Separate Coding of Different Gaze Directions in the Superior Temporal Sulcus and Inferior Parietal Lobule
Source: Curr Biol. 2007 Jan 9;17(1):20–5. doi: 10.1016/j.cub.2006.10.052 (PMC1885952; doi:10.1016/j.cub.2006.10.052)
Supplement: Document S1. Supplemental Results and One Table [file mmc1.pdf]

Supplemental Data

Separate Coding of Different Gaze Directions in the Superior Temporal Sulcus and Inferior Parietal Lobule

Andrew J. Calder, John D. Beaver, Joel S. Winston, Ray J. Dolan, Rob Jenkins, Evelyn Eger, and Richard N.A. Henson

Supplemental Results

Full Details of fMRI Acquisition and Analysis

A 3T Allegra system (Siemens, Erlangen, Germany) was used for acquiring 32 T2*-weighted sequential transverse echoplanar (EPI) images (64 × 64 3 × 3 mm^2 pixels, echo-time of 30 ms) per volume with blood oxygenation level-dependent (BOLD) contrast and a repetition time (TR) of 2080 ms. EPIs comprised 2-mm-thick axial slices taken every 3 mm, with a pitch of approximately 30° up at the front (so that eye-ghosting was minimized). The slices were positioned to encompass ventral and lateral temporal lobes and included the inferior parietal cortex (the inferior cerebellum and superior parietal cortex were not covered; see Figure 3 in the main text. One hundred eighty-eight volumes were acquired for the preadaptation phase, and two sessions of 590 volumes were acquired for the four adaptation phases. Discarding the first 5 volumes per session allowed for equilibration effects.

The fMRI data were analyzed with statistical parametric-mapping software (SPM2, www.fil.ion.ucl.ac.uk/spm2.html). Preprocessing of the image volumes included standard realignment and unwarping to correct for additional movement-by-susceptibility interactions, normalization to an EPI template in Talairach space from the Montreal Neurological Institute (MNI), and spatial smoothing by an 8 mm FWHM isotropic Gaussian kernel (note that the resulting stereotactical coordinates reported here are based on the Montreal Neurological Institute (MNI) brain and bear a close, but not exact, correspondence to the atlas of Talairach and Tournoux).

Responses to all experimental conditions were modeled by delta functions marking stimulus onset convolved with the canonical hemodynamic response function (HRF) to create the regressors of interest. Missed and incorrect responses were modeled as separate regressors. Voxel-wise parameter estimates for the regressors were obtained by maximum-likelihood estimation, with a temporal high-pass filter (cut-off 128 s) being used for removing low-frequency drifts and an AR(1) process being used for modeling temporal autocorrelation across scans [S1].

Images of contrasts of these parameter estimates were used in a second-level "group" analysis, in which subjects were treated as the only random effect. In the preadaptation phase, correct responses were contrasted (incorrect and missed responses were less than 5%). In the adaptation phase, the contrasts collapsed across correct and incorrect ("adapted") responses (the associated parameter estimates were weighted by the number of each). All data were analyzed in section 2 of the adaptation phase because, although a "direct" response to left gaze after left-gaze adaptation, for example, provides a behavioral index of adaptation, a correct "left" response cannot be interpreted as an absence of adaptation; a 10° left gaze may be perceived as being 5° left. For the adaptation phase, contrasts for each of the six conditions were entered into a general linear model, assuming a pooled error. Given the multiple measurements over voxels (unlike the single behavioural measure), the degree of nonsphericity could be estimated directly from the subset of "activated" voxels, allowing maximum likelihood estimates of the condition effects rather than ad hoc correction to the degrees of freedom (see Friston et al., 2002, Neuroimage, for further details). The critical, directional interaction between adapting gaze direction and probe gaze direction that was predicted a priori was evaluated as a T contrast within the full model, which created a statistical parametric map (SPM) of the T statistic. Effects were predicted for regions that human and nonhuman primate research has implicated in gaze perception; specifically, such regions are the STS and parietal cortex [S2–S10]. Because no standard anatomical ROIs are available for both a priori regions, we thresholded the SPMs at an a priori threshold of $p < 0.001$, uncorrected for multiple comparisons.

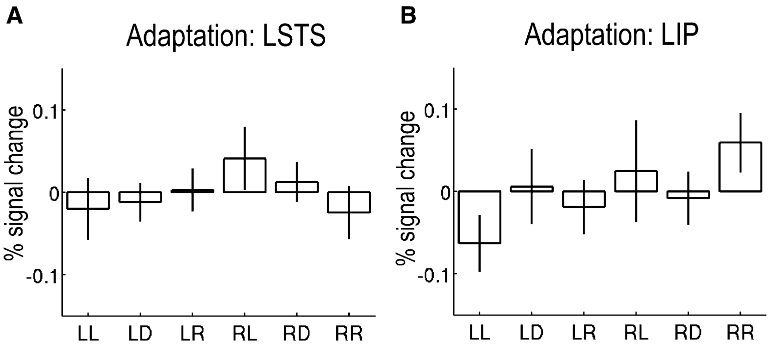

Figure S1. Neuroimaging Data

Mean event-related response to each of the three types of probe faces (10° left, direct, and 10° right) as a function of the direction of gaze adaptation (left and right) for two *left*-hemisphere voxels corresponding to "reflections" of (A) the maximally activated voxel in the right anterior STS (LSTS; −57, 9, −27), and (B) the maximally activated voxel in the right inferior parietal lobule (LIP; −60, −54, 30). The y axis represents estimated peak percent signal change relative to the average over all voxels and scans; error bars show standard error of the mean, between-subject differences having been removed. LL = left adaptation-left gaze probe; LD = left adaptation-direct gaze probe, and so on.

Table S1. Mean Percentage of "Left," "Direct," and "Right" Responses in the Preadaptation, Left-Adaptation, and Right-Adaptation Phases of the Experiment for the Behavioral Task

Direct Responses			
Preadaptation	3%	96%	7%
Left adaptation	65%	97%	8%
Right adaptation	6%	89%	74%
Left Responses			
Preadaptation	97%	2%	0%
Left adaptation	35%	0%	0%
Right adaptation	94%	11%	0%
Right Responses			
Preadaptation	0%	1%	93%
Left adaptation	0%	3%	92%
Right adaptation	0%	0%	25%

Analysis of Behavioral and Neuroimaging Data with ANOVA

In addition to the congruent versus incongruent (gaze adaptation/gaze probe) planned comparison reported in the main text, behavioral data and neuroimaging data were also analyzed with repeated-measures ANOVA for examination of the event-related response across all three types of probe faces (left, direct, and right gaze) as a function of the adapting gaze direction (left or right). Any effect of adaptation was therefore expressed as an interaction between these two factors.

Behavioral Data

The analysis of correct responses showed a significant interaction between adapting gaze direction and probe gaze face, $F(1.3, 16.5) = 126.19$, $p < 0.0001$, Greenhouse-Geisser corrected. t tests showed that after left-gaze adaptation, decreased correct responses were observed for left relative to right gaze probes, $t(13) = 10.36$, $p < 0.0001$; in contrast, right-gaze adaptation produced decreased correct responses to right-gaze probes, $t(13) = -9.93$, $p < 0.0001$.

Neuroimaging Data

After preprocessing, the estimated impulse response to each of the three types of probe faces (left, direct, and right) as a function of the adapting direction (left or right) was submitted to repeated measures ANOVA. This showed a significant interaction and identified the same two coordinates as the congruent versus incongruent contrast reported in the main text—right anterior STS (57, 9, −27; $F(1,65) = 14.96$ [$Z = 3.47$], $p < 0.0005$, nonsphericity adjusted), and right inferior parietal lobule (60, −54, 30; $F(1,65) = 12.41$ [$Z = 3.16$], $p < 0.0008$, nonsphericity adjusted). Further analyses of the maximum voxels' data revealed that the STS showed an effect of adaptation (when left and right probes were compared) after both left- (LL versus LR, $t(13) = -2.46$, $p = 0.014$) and right-adaptation (RR versus RL, $t(13) = 2.07$, $p = 0.029$) gaze-adaptation conditions. The same t tests for the inferior parietal region showed a similar pattern (LL versus LR, $t(13) = -3.44$, $p = 0.002$) and similar right-adaptation conditions (RR versus RL, $t(13) = 1.47$, $p = 0.082$).

The only suprathreshold voxels (i.e, the anterior STS and inferior parietal regions) were in the right hemisphere. To test for a laterality effect, we examined the responses of homologous regions in the left hemisphere (by inverting the sign of the x coordinate of the maximum voxel). The mean event-related responses from all four voxels (left STS, right STS, left inferior parietal, right inferior parietal) were entered into a repeated-measures ANOVA with factors hemisphere (left, right), region (STS, inferior parietal), adaptation direction (left, right), and probe direction (left, direct, right). This showed a significant three-way interaction between probe direction, adaptation, and hemisphere ($F(1.37,17.86) = 7.16$, $p = 0.01$); this interaction that was not qualified by a four-way interaction with region ($F < 1$). This confirmed the right lateralization of the present gaze-adaptation effects.

Supplemental References

S1. Friston, K.J., Glaser, D.E., Henson, R.N.A., Kiebel, S., Phillips, C., and Ashburner, J. (2002). Classical and Bayesian inference in neuroimaging: Applications. Neuroimage *16*, 484–512.

S2. Perrett, D.I., Smith, P.A.J., Potter, D.D., Mistlin, A.J., Head, A.S., Milner, A.D., and Jeeves, M.A. (1985). Visual cells in the temporal cortex sensitive to face view and gaze direction. Proc. R. Soc. Lond. B. Biol. Sci. *B223*, 293–317.

S3. Perrett, D.I., Hietanen, J.K., Oram, M.W., and Benson, P.J. (1992). Organization and functions of cells responsive to faces in the temporal cortex. Philos. Trans. R. Soc. Lond. B Biol. Sci. *B335*, 23–30.

S4. Hoffman, E.A., and Haxby, J.V. (2000). Distinct representations of eye gaze and identity in the distributed human neural system for face perception. Nat. Neurosci. *3*, 80–84.

S5. Puce, A., Allison, T., Bentin, S., Gore, J.C., and McCarthy, G. (1998). Temporal cortex activation in humans viewing eye and mouth movements. J. Neurosci. *18*, 2188–2199.

S6. Pelphrey, K.A., Morris, J.P., and McCarthy, G. (2005). Neural basis of eye gaze processing deficits in autism. Brain *128*, 1038–1048.

S7. Pelphrey, K.A., Viola, R.J., and McCarthy, G. (2004). When strangers pass: Processing of mutual and averted gaze in the superior temporal sulcus. Psychol. Sci. *15*, 598–603.

S8. De Souza, W.C., Eifuku, S., Tamaru, R., Nishijo, H., and Ono, T. (2005). Differential characteristics of face neuron responses within the anterior superior temporal sulcus of macaques. J. Neurophysiol. *94*, 1252–1266.

S9. Wicker, B., Michel, F., Henaff, M.A., and Decety, J. (1998). Brain regions involved in the perception of gaze: A PET study. Neuroimage *8*, 221–227.

S10. Grosbas, M.-H., Laird, A.R., and Paus, T. (2005). Cortical shifts involved in eye movements, shifts of attention, and gaze perception. Hum. Brain Mapp. *25*, 140–154.
